# MRD in Philadelphia Chromosome-Positive ALL: Methodologies and Clinical Implications

**DOI:** 10.1007/s11899-024-00736-9

**Published:** 2024-06-18

**Authors:** Valerie Tran, Kiarash Salafian, Kenan Michaels, Caroline Jones, Daniel Reed, Michael Keng, Firas El Chaer

**Affiliations:** 1 Division of Hematology and Oncology, Department of Medicine, The University of Virginia, Charlottesville, VA USA; 2 Department of Medicine, The University of Virginia, Charlottesville, VA USA

**Keywords:** Acute Lymphoblastic Leukemia, Philadelphia Positive, Measurable Residual Disease, Blinatumomab, Inotuzumab Ozogamicin, Tisagenlecleucel

## Abstract

**Purpose of Review:**

Measurable residual disease (MRD) is integral in the management of Philadelphia chromosome-positive (Ph+) acute lymphoblastic leukemia (ALL). This review discusses the current methods used to evaluate MRD as well as the interpretation, significance, and incorporation of MRD in current practice.

**Recent Findings:**

New molecular technologies have allowed the detection of MRD to levels as low as 10^− 6^. The most used techniques to evaluate MRD are multiparametric flow cytometry (MFC), quantitative reverse transcription polymerase chain reaction (RT-qPCR), and high-throughput next-generation sequencing (NGS). Each method varies in terms of advantages, disadvantages, and MRD sensitivity. MRD negativity after induction treatment and after allogeneic hematopoietic cell transplantation (HCT) is an important prognostic marker that has consistently been shown to be associated with improved outcomes. Blinatumomab, a new targeted therapy for Ph + ALL, demonstrates high efficacy in eradicating MRD and improving patient outcomes. In the relapsed/refractory setting, the use of inotuzumab ozogamicin and tisagenlecleucel has shown promise in eradicating MRD.

**Summary:**

The presence of MRD has become an important predictive measure in Ph + ALL. Current studies evaluate the use of MRD in treatment decisions, especially in expanding therapeutic options for Ph + ALL, including tyrosine kinase inhibitors, targeted antibody therapies, chimeric antigen receptor cell therapy, and HCT.

## Introduction

Major advances in genomics have changed the treatment landscape of Philadelphia chromosome-positive (Ph+) acute lymphoblastic leukemia (ALL) [1]. This disease is characterized by the BCR-ABL fusion protein encoded by translocation t(9;22)(q34;q11), which plays a central role in the development of Ph + ALL. This fusion protein is a key biomarker for diagnosing and monitoring Ph + ALL [2–4]. Over the past few years, the incorporation of measurable residual disease (MRD) testing has become the standard of care for evaluating and treating ALL [1]. The role of MRD is being studied extensively, especially in the context of an expanding therapeutic arsenal for this disease, including tyrosine kinase inhibitors (TKIs), allogeneic hematopoietic cell transplantation (HCT), targeted antibody therapies, and chimeric antigen receptor T (CAR-T) cell therapy [5–7]. This review discusses the different techniques used to measure MRD and their use in clinical practice to treat Ph + ALL.

## Techniques for Measuring MRD

Various techniques exist to evaluate and quantify MRD in Ph + ALL, each with its own advantages, disadvantages, sensitivity, and accessibility (Table 1). Those used most regularly in current clinical practice are multiparametric flow cytometry (MFC), quantitative reverse transcription polymerase chain reaction (RT-qPCR), and high-throughput next-generation sequencing (NGS) [4].

**Table 1:** Comparison of different MRD techniques for Ph chromosome-positive ALL

	MFC	RT-qPCR	RQ-PCR	ddPCR	NGS
**Turnaround Time**	3–4 h	2–3 days	2–3 weeks	5–8 h	1 week
**Cost per sample**	$	$$	$$	$$	$$$
**Advantages**	• Rapid • Relatively cheap • Does not require access to pre-treatment samples	• Sensitive • Does not require patient-specific primers	• Sensitive • Well-standardized	• Very sensitive	• Very sensitive • Fast • Uses standard primers
**Disadvantages**	• Lack of standardization • Requires fresh cells • Risk of false negative results from immunophenotypic shifts • Requires significant technical expertise	• Limited standardization	• Time intensive • Requires significant technical expertise • Requires access to pre-treatment tissue • Requires patient-specific primers	• Requires patient-specific primers • Limited availability • Limited efficacy data	• Not standardized • Limited access • Requires access to pre-treatment tissue • Complex process
**Sensitivity**	10^− 4^	10^− 4^ to 10^− 5^	10^− 4^ to 10^− 5^	10^− 6^	10^− 6^

Abbreviations: ALL: acute lymphoblastic leukemia; MFC, multiparametric flow cytometry; Ph, Philadelphia; RT-qPCR, quantitative reverse transcriptase polymerase chain reaction; RQ-PCR, real-time quantitative PCR; ddPCR, droplet digital PCR; NGS, next-generation sequencing

### Multiparametric Flow Cytometry

MFC has been central to the diagnosis of many hematopoietic malignancies for decades. At a fundamental level, this technology detects cellular subpopulations based on the expression of different antigens on individual cells. Samples on a flow cytometer are suspended in fluid and passed through a mechanism single file, exposing the samples to light of different wavelengths. The light then scatters in predictable ways based on the structural properties of the cells or specific fluorescent emission if tagged with certain fluorescent probe antibodies [8]. In the case of leukemias, including Ph + ALL, MFC relies on its ability to recognize cells that differ structurally from those that would be expected from similar cells of a specific lineage or stage of maturation. Non-malignant cells exhibit consistent, reliable patterns of antigen expression regardless of the age, sex, or race of the patient; therefore, through thoughtful fluorescent antibody combinations, MFC can differentiate leukemia-associated immunophenotypes (LAIPs) from benign [9, 10]. LAIPs on blast cells can be detected, tagged at diagnosis, and tracked throughout the treatment duration. The advantages of MFC over other MRD techniques exist mostly in the realm of logistics: the test has a fast turnaround time (generally less than four h), is nearly ubiquitously available at most medical centers, and is available at low cost. Unfortunately, these conveniences are limited by poor standardization, variable sensitivity, high false negative results, and the requirement for fresh samples (< 48 h old). While Europe has standardized its MFC-MRD assessment [11], the United States has not. This lack of standardization and variability is likely related to the significant expertise required by the pathologists, which can contribute to poor inter-laboratory reliability. MFC can reliably detect one leukemic cell per 10,000 cells [11]. MRD analysis by MFC in Ph + ALL and other leukemias has begun to fall out of favor with the advent of newer, more advanced technologies described below [12].

### Polymerase Chain Reaction

PCR plays a more central role in MRD within Ph + ALL, improving several aspects of MFC. Building upon the basic tenets of cell biology, PCR amplification of complementary strands of DNA allows for the exponential multiplication of genetic material to diagnose or monitor countless hematologic malignancies [13]. In contrast to MFC, where the techniques and molecular targets involved are similar between Ph + and Ph- ALL, PCR-based MRD testing differs because of the specific molecular targets for the Philadelphia chromosome. MRD in Ph + ALL primarily utilizes quantitative reverse transcription PCR (RT-qPCR), in which mRNA gene transcripts undergo reverse transcription to form cDNA exons and are subsequently amplified through PCR. The genes that the cells intended for protein production can then be targeted with probes and analyzed for the presence of fusion genes (such as BCR-ABL1) [14]. Other fusion genes have been targeted in other ALL subtypes, but there is little data to support the efficacy of tracking these genes for MRD purposes, irrespective of Philadelphia chromosome positivity [15]. This PCR method is uniquely suited for Ph + ALL and lacks utility in other leukemias, such as Ph- ALL, where real-time quantitative PCR (RQ-PCR) must be used because of the lack of fusion gene targets for reverse transcription [16].

RQ-PCR is based on the understanding that B- and T-cell progenitors undergo random somatic recombination of variable (V), diversity (D), and joining (J) gene segments of their immunoglobulin heavy chain (B-cells) and T-cell receptors (T-cells) during mitosis in the early stages of development. Leukemic transformation, typically after VDJ rearrangement, forms clonal immunoglobulin heavy chains and T-cell receptor rearrangement. At the junctional regions of these randomized genes, closest to where the most unique sequences occur [4], allele-specific oligonucleotides (ASOs) can be engineered and developed for each individual patient, which can be recycled and used throughout the treatment course to track MRD [17]. Although most studies on Ph + ALL use RT-qPCR to evaluate for MRD, some data indicate that RQ-PCR examining heavy chain and T-cell receptor rearrangements may actually be superior, as BCR-ABL1 can sometimes be detected in non-ALL hematopoietic cells causing an increase in false positive results [18]. Despite this, RT-qPCR for BCR-ABL1 fusion protein remains the most common method for MRD assessment in US academic centers [4].

Several studies have compared the two PCR methods in the Ph + ALL population and found that they are quite concordant (around 70%) and reliable at predicting outcomes [18]. However, it has rarely been demonstrated that patients with persistently positive BCL-ABL1 levels via RT-qPCR after treatment could actually be presenting with a “chronic myeloid leukemia (CML)-like” disease, which would be missed with standard RQ-PCR [19]. This “CML-like” disease phenotype is described below. Therefore, RT-qPCR analysis of fusion proteins and RQ-PCR for heavy chain/T-cell receptor rearrangement should be used in tandem for MRD detection in patients with Ph + ALL [20]. Both RT-qPCR and RQ-PCR have several advantages and limitations compared to MFC [4, 14, 16, 20]. Both are increasingly sensitive compared to MFC, with the ability to detect one leukemic cell in 10^5^ cells. RQ-PCR is also a well-standardized MRD technique for all ALL types through the EuroMRD consortium [21]. RT-qPCR is less standardized in terms of laboratory procedures and data interpretation in MRD; however, this has improved since 2019 through the efforts of Pfeiffer et al. [22]. These benefits of PCR come at the expense of time-intensive, costly, and labor-intensive processes, particularly with RQ-PCR. This is due to the expertise that pathologists and lab technicians require and the need for patient-specific primers to be developed, necessitating access to pre-treatment samples (not required with RT-qPCR). RT-qPCR benefits from the need for standardized fusion gene primers.

Discussing PCR in MRD for Ph + ALL would be incomplete without briefly discussing the relatively new emerging PCR technology termed droplet digital polymerase chain reaction (ddPCR) [23]. First demonstrated in 1999, ddPCR is performed by separating the reaction mixture into 20,000 droplets in microscopic wells; PCR amplification is then completed with the addition of fluorescent probes, as in RQ-PCR [24]. The presence or absence of fluorescence is then analyzed using a Poisson distribution, which allows for absolute DNA quantification in contrast to QR-PCR, which can only return relative quantification [25]. The benefits of ddPCR over normal RQ-PCR and RT-qPCR include increased sensitivity to detection (one blast per 10^6^ cells). Furthermore, recent data demonstrated that this increased sensitivity over standard PCR techniques translated into superior MRD evaluation, specifically in the setting of Ph + ALL, opening the door for this to conceivably be the recommended technique for MRD in adult BCR/ABL1 + ALL cases in the future [26, 27]. The technology has been commercially available since 2011 but lacks standardization due to its recent emergence as a viable, more sensitive alternative [14].

### Next-Generation Sequencing

NGS is the third and final distinct technology that has emerged in the MRD space. This addresses some of the shortcomings of traditional PCR and MFC. NGS uses multiplex PCR with universal (not requiring patient-specific) primers to amplify any possible immunoglobulin or T-cell receptor VDJ regions, similar to RQ-PCR but on a much larger scale and with significantly increased granularity [28]. Therefore, it can track and quantify various clones and subclones throughout the treatment duration, allowing for a highly efficient, effective, and reliable MRD technique [29, 30]. Similar to ddPCR, NGS allows the molecular detection of one leukemic cell in 10^6^ cells. With the correct amount of genetic material, sensitivities can be as high as 10^7^, but this quantity of substrate is rarely, if ever, obtainable in routine clinical practice [14, 31]. Nevertheless, high sensitivity to leukemic cells at the level of NGS allows for possible MRD quantification in the peripheral blood rather than bone marrow (where leukemic cell concentrations are at least 10-fold higher than those in peripheral blood) [32], potentially sparing patients from repeated bone marrow biopsies throughout the course of treatment. NGS is well standardized, and the FDA approved the first NGS technology in the US in 2018 [33]; however, further research is needed to fully elucidate the clinical significance of NGS’s increased sensitivities [20]. Early retrospective analyses have demonstrated an excellent correlation between the current MRD standard-of-care RT-qPCR and NGS assays, specifically in the Ph + ALL patient population [34], paving the way for future adoption as the foundation of MRD assessment. The drawbacks of NGS include cost and the need for access to pre-treatment specimens; turnaround time is no longer than that of its PCR counterparts.

## Practical Considerations

When assessing MRD in clinical practice, one must consider the practical applications of the techniques described above and the timing of when to re-sample for MRD.

### Source of Sample

A developing area of research within those methods with particularly high sensitivities for leukemic cells pertains to whether peripheral blood evaluation for MRD is adequate compared to a painful, time-intensive bone marrow sample. Most studies aiming to answer this question in ALL with RT-qPCR and MFC have not been performed specifically for Ph + ALL (instead only within Ph- ALL) [35, 36]. One small study from 1995 evaluated MRD using RT-qPCR in a sample of 18 Ph + ALL patients. MRD by RT-qPCR was detected in the marrow but not in the peripheral blood in four (22%) patients [37]. This study established that a bone marrow sample is superior to a peripheral blood sample and must be used to evaluate MRD with RT-qPCR in Ph + ALL. It should be noted that there is still some prognostic value of MRD in the peripheral blood by RT-qPCR, as patients found to have MRD in both the peripheral blood and bone marrow have been found to have a much higher risk of relapse than those with MRD in the bone marrow alone [35]. Since MFC and PCR require bone marrow sampling, NGS may help to change this paradigm. No studies we are yet aware of have specifically evaluated MRD assessment by NGS in the peripheral blood versus the bone marrow in Ph + ALL [4]. However, because NGS does not screen solely for fusion proteins (such as *BCR/ABL1*) and instead looks at the sum of all randomized VDJ IGH regions, it may be reasonable to extrapolate results comparing peripheral blood and bone marrow MRD evaluation in Ph- ALL. Logan et al. retrospectively examined MRD with NGS in ALL and found it capable of adequately predicting relapse and survival in post-transplantation patients [38]. Muffly et al. prospectively evaluated NGS-based MRD in adult ALL in 126 paired peripheral blood and bone marrow samples in the post-HCT/post-CAR-T setting and found excellent agreement between the sampling methods [32]. Pulsipher et al. retrospectively demonstrated impressive sensitivities of peripheral blood NGS compared to bone marrow MFC in children and young adults with ALL [5]. Further research with larger trials, specifically in the setting of Ph + ALL, is needed to further explore and validate these findings, and consensus guidelines continue to recommend sampling the bone marrow for MRD at a minimum [4, 39].

### Interpreting Discrepant Results

Interpretation of MRD can be further complicated by discordant results between the different forms of MRD assessment. Each study comparing various forms of MFC to PCR to NGS universally has at least some level of discrepancy [27, 34, 40]. Hence, it is the standard of care in Ph + ALL patients to send multiple types of MRD assessments simultaneously throughout the course of treatment. Due to the genetics of some types of Ph + ALL, the results of RT-qPCR and NGS/RQ-PCR lack agreement; this is usually demonstrated by a positive RT-qPCR for *BCR/ABL1* fusion protein but negative clonal VDJ rearrangement (by NGS and/or RQ-PCR) [34]. Termed “CML-like” due to the presence of what is likely the *BCR/ABL1* fusion protein in the stem cell or myeloid lineage (as opposed to the lymphoid lineage), the genetics of this positive RT-qPCR are unrelated to the active ALL that would usually benefit from the monitoring of the presence of the *BCR/ABL1* protein. Studies have demonstrated that the overall survival rates of “CML-like” and “typical” Ph + ALL are the same, but this is likely due to the toxicity of unnecessary ALL treatments in those who have CML biology as compared to the high risk of lethal relapse in typical ALL [41]. Patients with CML-like disease have also been shown to not benefit from HCT [42]. Thus, it will be increasingly important in the future to assess the presence of the *BCR/ABL1* protein outside the clonal ALL population when applicable and to ensure the simultaneous use of VDJ MRD assessment methods (NGS/RQ-PCR) in adult Ph + ALL to guide therapeutic decisions (i.e., RT-qPCR should never be used in isolation for MRD analysis in Ph + ALL).

### Timing of MRD Assessment

In regards to the timing of MRD assessments for Ph + ALL, consensus guidelines and prior clinical trials generally adhere to the following recommendations for when to first assess or re-assess for MRD: after induction, in early consolidation (after approximately 3 months of consolidation therapy), and then every three months thereafter for at least 5 years for those who do not undergo a stem cell transplant in the first remission [3, 4, 43, 44]. Those who undergo stem cell transplantation should have MRD assessed immediately before transplantation and then every three months in perpetuity [4]. If patients enter a period of relapse or refractory disease, MRD should be assessed in patients who reach morphological remission at the end of treatment [4]. The presence or absence of MRD at these time points allows adherence to the treatment algorithms outlined in clinical trials.

## Clinical Significance of MRD Monitoring

### Time to MRD Negativity and its Effect on Survival and Risk of Relapse

Although MRD has been extensively studied as a prognostic factor in Ph- ALL, little is known about the outcomes in Ph + ALL. However, several studies have shown the prognostic importance of achieving MRD negativity, particularly early in the course of treatment. Short et al. investigated outcomes in Ph + ALL patients receiving standard induction chemotherapy plus tyrosine kinase inhibitor (TKI) with TKI maintenance thereafter, without undergoing HCT [45]. Their group found that patients who achieved MRD negativity at 3 months had significantly improved median overall survival (OS) and relapse-free survival (RFS) compared to those with a lower response (127 months versus 38 months; *P* = 0.009; and 126 versus 18 months; *P* = 0.007, respectively) [45]. Ravandi et al. studied a similar group of patients receiving conventional chemotherapy plus TKI without HSCT and found that patients who were negative for MRD at 3 and 12 months post-induction had significantly longer survival and complete remission (CR) duration [40]. Additionally, obtaining a major molecular response (MMR) or better at 3, 6, 9, and 12 months was significantly associated with improved survival, although this did not reach statistical significance for CR duration [40]. Daver et al. also showed significantly improved disease-free survival (DFS) in patients treated with hyper-CVAD and imatinib who achieved MMR or CMR at 3 months [46]. Both Short’s and Ravandi’s groups found that achieving MRD negativity at CR did not affect OS, suggesting that obtaining a durable negative MRD response during one’s treatment course may be more critical in predicting superior outcomes.

Achieving MRD early in treatment also appears to predict better outcomes post-HCT, although the data are mixed. Lee et al. studied MRD kinetics on long-term HCT outcomes and determined that early molecular responders (patients achieving a major or complete molecular response by the end of two courses of imatinib-based chemotherapy) were significantly less likely to relapse post-transplant and had a significantly greater DFS than late, intermediate, and poor molecular responders [6]. However, MRD levels did not retain statistical significance in multivariate analysis after the first chemotherapy course. Yanada et al. also studied the effect of MRD level on treatment outcomes in patients with Ph + ALL treated with imatinib combined with chemotherapy and showed that negative MRD at the end of induction therapy was not associated with longer RFS or a lower relapse rate [47]. However, this study included non-transplant and transplant patients, which may explain the different results.

### MRD Effect on Allogeneic HCT Outcomes

While longer remissions have been achieved with TKI plus chemotherapy, -HCT remains the only treatment with definitive curative potential [48, 49]. Recently, several studies have shown that MRD status at the time of HCT can predict survival outcomes and risk of relapse. In a prospective analysis, Lussana et al. found that patients achieving pre-transplant MRD negativity were significantly less likely to have relapsed at 5 years post-transplant, compared to those with MRD positivity (relapse incidence of 8% versus 39%, respectively; *P* = 0.007) [49]. Their group did not find significantly different 5-year disease-free survival (DFS) and overall survival (OS) probabilities between MRD-negative and MRD-positive patients, though significantly more MRD-positive patients received post-transplantation TKIs and/or donor-lymphocyte infusions (DLI) compared to MRD-negative patients (61% vs. 33%, *P* = 0.031). In a larger retrospective analysis, Nishiwaki et al. showed that patients transplanted with MRD-negative disease had significantly improved 4-year OS and had a significantly lower incidence of relapse than patients transplanted with MRD-positive disease [50]. Mizuta et al. similarly showed a significantly lower incidence of relapse at 3 years post-HCT in MRD-negative transplanted patients than in MRD-positive patients but did not find a significant difference in non-relapse mortality (NRM) [51].

## Treatment Considerations

### Blinatumomab in MRD-Positive Disease

In recent years, the development of targeted therapies, such as blinatumomab, has only further improved outcomes in patients with B-cell ALL, with the BLAST and ECOG-ACRIN E1910 trials showing improved survival in MRD + and MRD- patients, respectively [52, 53]. However, these trials almost entirely focused on patients with Ph- ALL. The ALCANTARA study evaluated blinatumomab monotherapy in patients with Ph + ALL who had relapsed or were refractory to at least one TKI [54, 55]. Their group found an overall response rate of 35.6%, but a complete MRD response was observed in 87.5% of patients among responders [55].

Following the positive results of multiple retrospective analyses investigating blinatumomab plus TKIs in relapsed/refractory Ph + ALL, the D-ALBA trial studied the effects of blinatumomab consolidation following dasatinib induction in patients with newly diagnosed Ph + ALL [56–60]. The molecular response increased from 29% (at the end of dasatinib induction) to 60% (after two cycles of blinatumomab) [60]. This response rate increased further (up to 81%) with additional cycles of blinatumomab, ultimately leading to robust survival rates, with an OS of 95% and DFS of 88% at a median follow-up of 18 months and an estimated 4-year overall survival of 78% [60, 61]. Among patients achieving a molecular response upon induction, DFS was 100% at 48 months (versus 67% in patients not achieving a molecular response, *p* = 0.016) [61]. Jabbour et al. studied the frontline concurrent administration of ponatinib and blinatumomab in patients with newly diagnosed, relapsed, or refractory Ph + ALL or CML in the lymphoid blast phase [62]. Of the patients with relapsed or refractory Ph + ALL, 85% achieved MRD negativity after one cycle of combination therapy, with an additional increase to 93% after two cycles [62]. However, those patients who did not subsequently undergo HCT (54% of the relapsed or refractory patient cohort) later relapsed or died (71%). Despite the poor prognosis that MRD positivity confers, the aforementioned studies illustrate the efficacy of targeted therapies, such as blinatumomab, in eradicating MRD and improving patient outcomes.

## Implications in Relapsed/Refractory Disease

### Inotuzumab

Inotuzumab ozogamicin is an anti-CD22 monoclonal antibody conjugated to the cytotoxic agent calicheamicin and has recently been shown to be effective in relapsed/refractory ALL. In the phase 3 INO-VATE trial, patients treated with inotuzumab had significantly higher complete remission rates than those in the standard therapy group [63]. However, for patients with Ph + disease, there was no significant difference in the rates of complete remission [63]. Among Ph + patients who did respond, significantly more patients treated with inotuzumab achieved MRD negativity compared to Ph + treated with standard of care (81% versus 33%, *p* = 0.009) [64, 65]. In a pooled retrospective analysis of the INO-VATE trial and the phase 1/2 study 1010, which also investigated inotuzumab in patients with relapsed/refractory ALL, Stock et al. specifically analyzed outcomes in Ph + ALL patients enrolled in these studies [64–66]. Similar to the INO-VATE trial, Study 1010 found that a considerable proportion (100%) of responding patients treated with inotuzumab achieved MRD negativity [64, 66]. Because of the high rates of MRD negativity in responding patients treated with inotuzumab, Stock et al. found that approximately twice as many inotuzumab patients were able to proceed with HCT as compared to the standard of care group (41% vs. 19%) [64, 65]. This considerable increase allows more patients with Ph + ALL to undergo HCT and potentially cure their disease.

### Tisagenlecleucel

Given their ability to target specific tumor antigens, CAR T-cells have emerged as a promising novel therapeutic strategy for treating certain leukemias. As the CD-19 antigen is commonly expressed in most B-cell malignancies, CD-19-specific CAR T-cell therapy has recently been shown to be an effective treatment option for ALL. Maude et. al’s pilot phase I/IIA study investigated the anti-CD19 CAR T-cell therapy Tisagenlecleucel in 30 children and adults with relapsed/refractory ALL (not Ph + specific) and found a CR rate of 90%, with 19 patients having sustained remissions (follow-up period of 2–24 months) [67]. Notably, 81% of the responding patients achieved MRD negativity. The follow-up phase II ELIANA trial of 75 patients with CD19 + relapsed/refractory ALL again showed a significant treatment response to tisagenlecleucel, with an overall response rate of 81% [68]. In addition, all the responders were MRD-negative. Laetsch et al. expanded upon these results in the three-year update of the ELIANA trial and found that patients treated with tisagenlecleucel had an overall remission rate of 82% with a median EFS of 24 months and median OS that was not reached [69]. At the 3-year follow-up, the EFS was 44%, and the OS was 63% [69]. All but one of the responding patients had MRD-negative disease. Patients with Ph + ALL were enrolled in these trials, but a specific subgroup analysis was not performed on these patients [2].

Similarly, the phase 2 ZUMA-3 trial studying another autologous anti-CD19 CAR T-cell therapy, Brexucabtagene autoleucel (KTE-X19), in 71 adult patients with relapsed/refractory B-cell ALL, showed a significant treatment response [70]. Of the 55 patients receiving KTE-X19, 71% achieved CR or complete remission with incomplete hematological recovery (CRi), with 56% achieving CR. Among the responders, 97% had MRD-negative disease [70]. Shah et al.’s two-year follow-up analysis showed median remission and OS durations of 14.6 months and 25.4 months, respectively, at a median follow-up of 26.8 months [71]. The MRD negativity rate among responders remained at 97%. Notably, 27% of the patients had Ph + ALL. The SCHOLAR-3 retrospective historical control study built upon the ZUMA-3 data and evaluated the outcomes of ZUMA-3 patients matched to adult patients with R/R B-cell ALL treated in historical clinical trials. Brexucabtagene autoleucel proved to have a robust treatment effect with a median OS of 25.4 months compared to 5.5 months for matched historical controls [71, 72]. While both tisagenlecleucel and brexucabtagene autoleucel have been shown to significantly improve patient outcomes in this particularly difficult-to-treat patient population with relapsed/refractory ALL, evidence regarding their efficacy in Ph + ALL remains limited and highlights a need for further investigation.

### Impact of MRD on the Decision to Pursue HCT

Given the improvement in the depth and durability of treatment responses, there is increasing interest in sparing patients possible transplants and their associated toxicities. Nishiwaki et al. analyzed three prospective clinical studies that used either imatinib or dasatinib for Ph + ALL and investigated how HCT in CR1 with complete molecular remission affected the outcomes in this population. The study demonstrated both superior OS (aHR, 0.54 [95% CI, 0.30–0.97]; *p* = 0.04) and RFS (aHR, 0.21 [95% CI, 0.12–0.38]; *p* < 0.01) compared to those that did not undergo HCT [73] This study suggests the importance of HCT in the TKI era for Ph + ALL.

The GIMEMA LAL2217 ancillary trial of the D-ALBA study showed prolonged survival in patients maintained solely with TKI therapy after induction with dasatinib plus steroids followed by blinatumomab consolidation [74]. Nearly half of the study population did not receive chemotherapy or transplant, given that nearly all of these patients (93.3%) achieved significant molecular response after dasatinib/blinatumomab [74]. All but one remained in complete hematologic response (CHR) at 4 years. Most patients who received HCT in the first CHR were MRD-positive but still achieved substantial treatment benefits, as 83.3% were alive in CHR at a median follow-up of 49 months [74]. These results illustrate that early molecular responders can remain in sustained remission without the need for a transplant, whereas HCT still serves as an effective treatment for MRD-positive patients.

The GRAAPH-2005 multicenter study compared outcomes in newly diagnosed patients with Ph + ALL who were treated with high-dose imatinib plus reduced-intensity chemotherapy versus standard imatinib with hyperCVAD, focusing on the major molecular response after cycle 2 in the hope of becoming eligible for possible HCT. While their group found that patients receiving HCT in CR1 had both improved RFS (HR, 0.69 [95% CI, 0.49–0.98]; *p* = 0.036) and OS (HR, 0.64 [95% CI, 0.44–0.93]; *p* = 0.02), there was no benefit from HCT in terms of RFS in patients with molecular CR (HR, 1.02 [95% CI, 0.47–2.21]; *p* = 0.96) [75]. Instead, those who remained MRD-positive did have significantly improved RFS with HCT (HR, 0.62 [95% CI, 0.40–0.96]; *p* = 0.034) [75].

Ghobadi et al. retrospectively analyzed outcomes in patients with Ph + ALL who achieved a complete molecular response (CMR) within 90 days of diagnosis. They observed no improvement in OS or RFS in patients undergoing HCT compared to those who did not receive HCT [76]. Despite a decrease in the incidence of relapse, patients treated with HCT had significantly higher non-relapse mortality (adjusted hazard ratio [aHR]: 2.59; 95% CI, 1.37–4.89; *p* = 0.003), likely accounting for the similar survival outcomes between the two cohorts. Sasaki et al. similarly found no difference in PFS or OS in patients undergoing HCT who achieved a complete molecular response after 3 months of TKI therapy [77]. With the advent of TKIs and targeted therapies, such as blinatumomab, patients are obtaining deeper molecular responses and better survival outcomes. As a result, an increasing number of patients may be able to have their disease controlled or managed entirely without the need for systemic chemotherapy and transplantation and their associated toxicities [78].

## Conclusion

MRD has become an important tool for stratifying and prognosticating patients with Ph + ALL. Numerous studies suggest that MRD affects OS and other outcome measures and validate MRD as a predictive measure in this disease. Although the timing of MRD evaluation varies from study to study, early MRD negativity has consistently been shown to be correlated with improved outcomes.

The benefits of MRD are dependent on the technologies used to evaluate it. MFC, RT-qPCR, and NGS are the most commonly used techniques for this purpose. The sensitivities of these methods have improved over time, allowing MRD detection to levels as low as 10^− 6^. Improved standardization of these techniques and improved availability and cost will allow for their widespread use in clinical practice.

Despite several advancements in targeted therapies for patients with Ph + ALL, a substantial proportion of patients will eventually have their disease relapse owing to the presence of MRD. This disease’s rapidly evolving therapeutic landscape warrants further investigation of its treatment implications related to MRD. Several studies discussed in this review have demonstrated the impact of MRD on determining the timing of HCT based on the MRD status, especially with the incorporation of new targeted therapies such as blinatumomab in the upfront setting. Future studies must elucidate the need for HCT in all patients who achieve MRD negativity in CR1. As such, an important implication of MRD in acute leukemias, including Ph + ALL, is its use in clinicians’ decisions to de-escalate or intensify therapies.

## Data Availability

No datasets were generated or analysed during the current study.
